# Toward a Unified Social Motor Cognition Theory of Understanding Mirror-Touch Synaesthesia

**DOI:** 10.3389/fnhum.2016.00246

**Published:** 2016-05-31

**Authors:** Shenbing Kuang

**Affiliations:** State Key Laboratory of Brain and Cognitive Science, Institute of Psychology, Chinese Academy of SciencesBeijing, China

**Keywords:** mirror-touch synaesthsia, mirror neuron system, mentalizing system, social motor cognition, predictive coding, common coding theory

Mirror-touch synaesthesia (MTS) is a conscious tactile sensation in the observer when watching somebody else being touched. Two disparate theories have been suggested to explain MTS. The threshold theory links MTS to hyper-activity in the parietal-frontal mirror neuron system, while the self-other theory attributes MTS to impaired self-other representations in temporal-parietal junction (TPJ) and medial prefrontal cortex (mPFC). Here, I propose that these two theories can be synthesized under a unified social motor cognition theory which states that action observation engages two complementary levels of cognitive processing: a lower-level, physical process regarding basic sensory-motor aspects of the action, which supports motor imitation and goal understanding, and an abstract mental level concerning attribution of mental states, which supports inferring others' minds and self-other distinctions. While the physical process preferentially recruits the mirror neuron system, the mental process depends critically on the mentalizing network comprised of TPJ and mPFC. Importantly, despite of these anatomical and functional dissimilarities, the mirroring and mentalizing processes involve shared predictive coding, which is a general computational principle for a wide range of prominent concepts in motor cognition.

## Introduction

Mirror-touch synaesthesia (MTS) is a special kind of tactile sensation in one's own body when seeing someone else being touched (Blakemore et al., 2005). While MTS people constitute only a minority (1.6%), studying the neural underpinnings of MTS provides important insights into the mechanisms of sensory, motor, and social cognitive functions in the brain. Although MTS has drawn increasing attention from the field of psychology and neuroscience in recent years, the underlying mechanisms remain largely controversial. As summarized recently in Ward and Banissy (2015), two different theories have been put forward to account for MTS. The threshold theory posits that MTS synaesthetes exhibit hyper-activity in the mirror neuron system, which leads to heightened somatosensory activation crossing certain perceptual threshold (Blakemore et al., 2005; Bolognini et al., 2013). The self-other theory claims that MTS is associated with impaired ability to distinguish self from others in temporal-parietal junction (TPJ) and medial prefrontal cortex (mPFC) (Banissy and Ward, 2013; Holle et al., 2013).

As mentioned in passing by Ward and Banissy (2015), the two theories are not necessarily mutually exclusive. Yet, it fails to offer specific explanations and/or speculations as to how the two disparate theories might be related. Here, I propose a unified social motor cognition theory which not only conceptually incorporates these two theories, but also potentially serves as a coherent, parsimonious interpretation for a broader range of prominent action cognition concepts that are closely related to MTS. Below, I will first address the concept that the threshold theory and the self-other theory reflect nothing but two complementary levels of cognitive processing during action observation. I will then elaborate and discuss the shared neural codes and computational principles between these two cognitive processes.

## Complementary processing during action observation: mirroring and mentalizing

Contemporary view in social motor cognition holds that action observation triggers two different levels of cognitive processing which are supported by distinct brain systems (De Lange et al., 2008; Van Overwalle and Baetens, 2009). The lower-level, physical processing concerns basic sensory-motor and kinematic representations, which are good for motor imitation and for prediction of sensory outcome of an observed action to facilitate goal understanding (Rizzolatti and Sinigaglia, 2010). It is generally accepted that the physical mirroring aspects of action recognition are supported by the mirror neuron system, located primarily in the frontal-parietal circuits comprised of ventral premotor cortex, dorsal premotor cortex, and anterior intraparietal sulcus (Gallese and Goldman, 1998). In contrast, the higher-level, abstract mentalizing process involves attributing mental states (thoughts, desires, intention, etc) to oneself and to others, which supports inferring others' minds, self-awareness, and self-other distinctions (Frith and Frith, 2006; Lieberman, 2007). The mentalizing process engages a distinct set of brain networks mainly including area TPJ, area mPFC, and posterior superior temporal sulcus (Amodio and Frith, 2006; Van Overwalle and Baetens, 2009). The mirroring and mentalizing systems are two anatomically distinct yet functionally complementary aspects of action recognition during social interactions (Mainieri et al., 2013; Spunt and Lieberman, 2013; Ciaramidaro et al., 2014; Sperduti et al., 2014).

The mirroring and mentalizing systems are often differentially recruited, depending on specific task demands and social-cognitive contexts. For instance, it has been shown that participants show increased activations in the mentalizing system when thinking about why an action in a video clip was performed, comparing to thinking about what the action was and how the action was performed (Spunt et al., 2011). Similarly, observations of familiar actions that have pre-existing sensory and motor repertoires preferentially activate the mirroring neuron system (Calvo-Merino et al., 2005), while observations of unfamiliar actions more strongly recruit the mentalizing network, probably reflecting the increased demand of mental inferences in order to make sense of novel actions (Brass et al., 2007). As such, the threshold theory for MTS likely reflects abnormal processing at a mirroring level, while the self-other theory corresponds to atypical representations at a mental level. In this way, the two competing theories are not separate theories for explaining MTS. Instead, they should be viewed as reflecting two complementary aspects of cognitive processes during touch observation, which work synergistically to ensure appropriate social interactions in a given behavioral context.

The dichotomy between the mirroring and mentalizing processes captures not only the threshold theory and the self-other theory, but also may explain a few additional concepts associated with MTS. For instance, the dichotomy suggests that self-other distinction should operate at both the mental and physical levels: the former refers to psychologically separating oneself from others and plays a role in self-awareness (Jenkins and Mitchell, 2011) and empathy (Decety and Jackson, 2004), while the latter supports several aspects of bodily self-consciousness (Ionta et al., 2011a; Blanke et al., 2014), which includes senses of body ownership (Tsakiris et al., 2007), sense of agency (Jeannerod, 2003; Jackson and Decety, 2004), as well as processing related to self-location and first-person perspective (Ionta et al., 2011b). Interestingly, people with MTS often exhibit various aspects of these anomalous self-experience at both the mental and physical body levels (Ward and Banissy, 2015), which can be parsimoniously interpreted as aberrant representations in the mirroring and mentalizing systems during touch observation.

## Shared coding principles between mirroring and mentalizing: predictive coding

The mirroring and mentalizing systems might have shared predictive coding principle. First, at the physical mirroring level, goal understanding, sense of agency, and bodily self awareness are each associated with predictive processing. Goal understanding hinges on the ability to make predictions about the consequence and sensory outcome of an observed action (Kilner et al., 2007). This prediction is thought to be based on efference copies of the mapped motor representations in the observer during action observation (Gallese and Goldman, 1998; Rizzolatti and Sinigaglia, 2010). Sense of agency depends critically on the congruency between the predicted sensory outcome and the actual sensory feedback associated with an action (Tsakiris et al., 2007). In a similar vein, a predictive coding account of bodily self awareness (Apps and Tsakiris, 2014) proposes that, recognizing one's self is a probabilistic process of multimodal integration between the actual sensory states (re-afference) and other bodily related information including predictions based on corollary discharge (efference). Second, at the abstract mentalizing level, theory of mind engages simulations of one's own intentions, desires, and beliefs to predict the mental states of others. This allows an individual to understand and empathize with others (Decety and Jackson, 2004). Our brain may be constantly making predictions at distinct levels during action observation, and deficits in these predictive processing will result in social-cognitive abnormalities such as MTS and neuropsychiatric symptoms including autism spectrum disorders (Van Boxtel and Lu, 2013) and schizophrenia (Biedermann et al., 2012).

Predictive coding is not restricted to social-cognitive processing. Instead, it is considered to be a general coding principle which underlies a wide variety of perceptual and motor functions (Brown and Brune, 2012). Take the field of motor cognition as an example, predictive coding has been well-established as the core principle for several prominent concepts. For instance, it serves as the underlying mechanism for adaptive motor control (Shadmehr et al., 2010; Franklin and Wolpert, 2011) and motor awareness (Blakemore and Frith, 2003; Desmurget and Sirigu, 2009), both of which involve internal forward predictions of sensory consequence of executed actions. It should be noted that, while forward models of action are framed in a predictive scheme, the underlying mechanisms involve neural computations specifically related to efference copy signals (or “corollary discharge”), which are different from predictive computations implemented in other brain functions such as visual processing (Rao and Ballard, 1999), associative learning (Schultz and Dickinson, 2000), and decision making (Rushworth et al., 2009). In addition to action execution, predictive coding has also been linked to concepts related to action selection and planning in a recent neurophysiology study (Kuang et al., 2016). It is shown that when monkeys are planning an arm movement, neurons in posterior parietal cortex encode not only the intended physical movement but also the visual sensory anticipation of the planned movement. These predictive coding of planned action support the longstanding ideomotor theory in cognitive psychology, which states that actions are planned and selected with respect to their perceptual consequences (Shin et al., 2010; Waszak et al., 2012). More broadly, the co-existence of physical and visual predictive representations in the same brain area is very reminiscent of the idea of common coding theory which posits a tight bi-directional link between action and perception systems (Prinz, 1987; Hommel et al., 2001).

## Conclusion

In summary, this paper provides a synthetic view for understanding MTS from the perspective of a unified social motor cognition theory. Instead of two competing, disparate theories, I propose that MTS is attributable to the disturbed mirroring and mentalizing functions, which represent the dual complementary aspects of cognitive processing with shared predictive coding during touch observation. Thus, the current unified viewpoint may serve as a coherent guiding principle for explaining diverse aspects of bodily and mentally abnormal phenomena in MTS populations.

## Author contributions

The author confirms being the sole contributor of this work and approved it for publication.

### Conflict of interest statement

The author declares that the research was conducted in the absence of any commercial or financial relationships that could be construed as a potential conflict of interest.
